# Construction and Operation of a High-Speed, High-Precision Eye Tracker for Tight Stimulus Synchronization and Real-Time Gaze Monitoring in Human and Animal Subjects

**DOI:** 10.3389/fnsys.2016.00073

**Published:** 2016-09-14

**Authors:** Reza Farivar, Danny Michaud-Landry

**Affiliations:** ^1^Department of Ophthalmology, McGill Vision Research Unit, McGill UniversityMontreal, QC, Canada; ^2^Research Institute of the McGill University Health CentreMontreal, QC, Canada

**Keywords:** eye tracker, saccades, oculomotor control, smooth pursuit, macaque, infant, gaze contingent, microsaccades

## Abstract

Measurements of the fast and precise movements of the eye—critical to many vision, oculomotor, and animal behavior studies—can be made non-invasively by video oculography. The protocol here describes the construction and operation of a research-grade video oculography system with ~0.1° precision over the full typical viewing range at over 450 Hz with tight synchronization with stimulus onset. The protocol consists of three stages: (1) system assembly, (2) calibration for both cooperative, and for minimally cooperative subjects (e.g., animals or infants), and (3) gaze monitoring and recording.

## Introduction

Eye tracking refers to the group of methods used to monitor oculomotor behavior, including the direction of gaze, spatio-temporal aspects of saccades, smooth pursuit, and fixational eye movements. Eye tracking measurements are important to many fields including neuroscience, psychology, marketing, and even political science. Performance of the eye tracking system restricts the kinds of questions that can be answered—while coarse measures of gaze direction can be carried out with about 1° of spatial resolution and 30–60 Hz sampling, measuring saccade latency accurately requires at least 250 Hz temporal sampling and detection of microsaccades requires resolution of finer than 20 arcmins (Poletti and Rucci, 2016).

High performance eye tracking systems with such specifications are typically prohibitively expensive (upwards of $30,000 USD), thus drastically limiting the number of laboratories that can include these essential behavioral measures in their research or the number of setups that can exist in one lab. Below we describe a protocol for the construction and use of a high-performance research grade eye tracking system that we call the McGill Vision Research Tracker (MVR Tracker).

The hardware design allows for tracking the eyes at a rate of more than 450 Hz and with a uniform precision of approximately 0.1° over the full typical viewing range of a computer monitor, while also allowing for precise alignment of the stimulus onset with the eye tracking data, thus enabling accurate stimulus-induced saccade latency estimates. The components of the eye tracking system altogether cost less than $1500 USD, greatly widening the availability of a research-grade eye tracking system for visual perception and oculomotor control studies.

### Applications of the method

The protocol is suitable for eye tracking in both cooperative and minimally cooperative subjects such as animals (monkeys, dogs, rats) or human infants. The system can operate at a lower sampling rate to either use a higher spatial sampling rate or operate on older computer systems.

### Comparison with other methods

High-performance eye tracking is commonly carried out with commercial tracking systems and there are no detailed protocols for assembling and operating a high-performance system that would also allow precise time stamping of stimulus events in cooperative and minimally-cooperative subjects. As seen below, the MVR Tracker performs comparably to many high-end commercial eye trackers.

### Level of expertise needed to implement the protocol

The protocol can be carried out by an undergraduate or a graduate student. The most complex part of the protocol involves soldering cables to connectors. If the lab does not have access to a soldering iron, the connections can be easily made with soldering glue (e.g., “Wire glue” or silver conductive glue, available for purchase through major online retailers). The majority of the protocol steps involve simply assembling, such as assembling lenses, filters, lens mounts, camera tripods, and the installation of the software on a computer.

### Limitations

The protocol has been successfully employed with (i) minimally-cooperative subjects (macaques) learning a visual psychophysics task, and responding by directing their saccade as recorded by the system, (ii) cooperative subjects carrying out a psychophysical task on an office desk, and (iii) subjects undergoing Magnetoencephalography (MEG) while having their eyes tracked with no observable artifacts on the MEG traces. In general, the system requires a stable head position, although it can tolerate small head displacement since it records the corneal reflection as well as the pupil position.

## Overview of protocol

**Figure 1:**
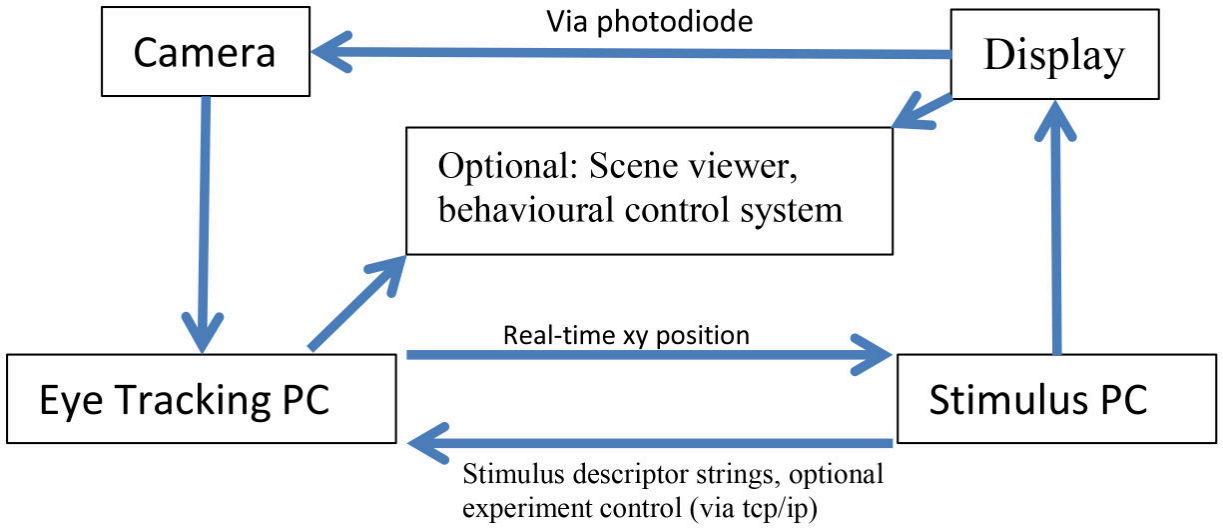
**Schematic layout of the connections of the MVR system, a stimulus PC, and an optional scene viewer or behavioral control system**. The interaction between the computers is via UDP and TCP/IP. Specifically, multiple computers can read the eye position from the tracker using UDP, while string descriptors for stimulus events can be sent to the eye tracker via TCP/IP, along with start and stop commands to control recording. The precise timestamping of the frame utilizes a photodiode that is triggered by a change in the display that then sends a TTL pulse to the camera which is registered in the image header acquired at that time point. The subject display can be viewed via a third computer.

**Figure 2:**
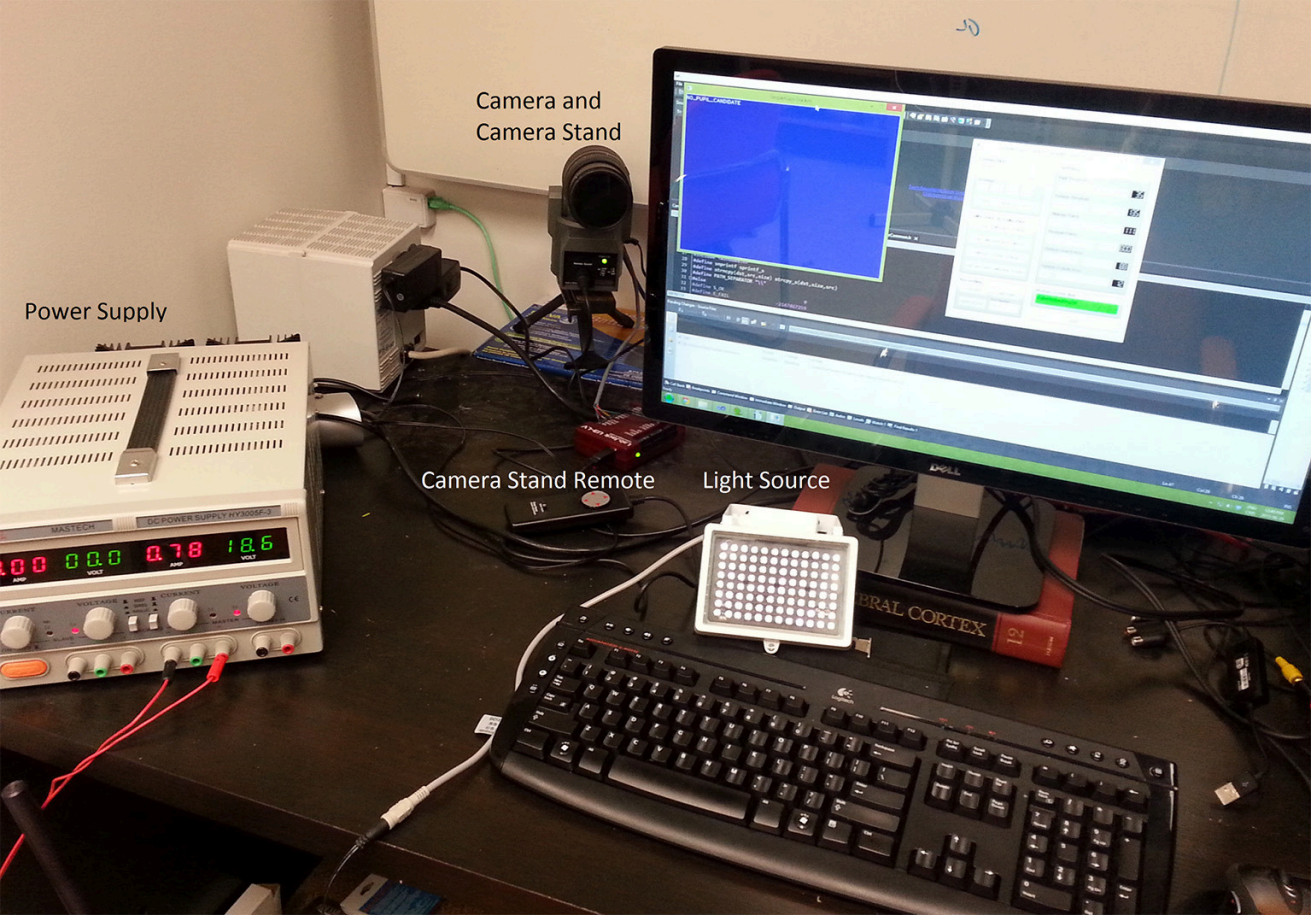
**A sample setup of the MVR tracker on a small student desk**. The MVR tracker consists of a regulated power supply to drive the light source at a constant illumination, a high-speed camera, and SLR optics mounted on a motorized pan-tilt camera stand. Optional features such as a photodiode for synchronization and scene viewer are not shown. The power supply can be placed away from the system.

Figure 1 describes the layout of the relationship between the MVR tracker, the stimulus PC, the stimulus display, and an optional scene viewer or behavioral reward control system. Figure 2 is a photo of the MVR tracker setup on a small student desk, depicting the key components.

### High-performance camera for acquisition of eye image

The key component of any video-based eye tracking system is the camera. We chose the Flea3 camera (model Flea3 FL3-U3-13Y3M-C) from Point Grey Research Inc. (Richmond, BC, Canada; ptgrey.com). This USB3.0 camera is capable of acquiring 640 × 480 images at approximately 490 frames per second (fps), which in our system yielded fixation traces with a standard deviation of ~0.1°. This camera features a global shutter, which means all pixels in one frame are acquired simultaneously, as opposed to the progressive shutters that are more commonplace in consumer-grade cameras that scan the sensor and acquire pixels sequentially (Point Grey Research Inc., 2015). Global shutter is critical for accurate tracking, because with progressive shutter methods, the eye image can be distorted in a given frame, introducing additional noise during eye movements.

Stimulus synchronization is critical in oculomotor studies, and this camera allows the researcher to input stimulus onset or other events directly into the camera. These stimulus events are then sampled at the exact same time as the image and with no latency, yielding frame-precise alignment of external events. Below we describe a simple system for synchronizing the onset of a stimulus display frame with the eye trace, but other external events can also be synchronized. The external event must be a standard TTL pulse and it is inputted to the camera via the GPIO connection. In the “Equipment setup” section we describe a simple circuit for detecting a stimulus onset on a computer monitor and synchronizing it directly with the eye trace.

### Optical components: lenses and lens mounts

Commercial eye trackers utilize the miniature c-mount lens system that is common in industrial systems. C-mount lenses have the advantage of being compact and can be inexpensive for certain applications where focal length is fixed. C-mount lenses are far smaller than lenses used in professional photography (single-lens reflex systems, or SLR). For this reason, c-mount lenses almost universally suffer from spherical aberration artifacts—a radial distortion and smearing of the image especially pronounced along the edges of the camera image due to the spatially non-linear refraction of light along the width of the lens. Spherical aberrations result in non-uniform accuracy during tracking—the image of the pupil can be badly distorted, especially during wide gaze changes to the edge of the tracking range. Spherical aberrations can either be corrected through use of more expensive aspheric lenses, or can be avoided through the use of a lens that is much wider than the camera sensor. With a larger lens, the entire field-of-view of the sensor falls on the central (linear) portion of the lens and thus spherical aberrations are mitigated. Larger lenses have the additional feature of allowing for more light on the sensor.

Our setup uses prosumer-grade SLR lenses that are widely available. We chose an economical lens with a 70–300 mm zoom range, which provides for great flexibility in the positioning of the camera with regards to the subject, as well as for increasing the portion of the image covering the pupil—for greater spatial precision in tracking. The zoom lens can easily be converted into a zoom macro lens (i.e., focused on a nearby zoomed object) by use of close-up lenses that are mounted on the screw-mount of the SLR lens. These close-up lenses are widely available for practically all lens sizes and formats. We used a +3 (or combination of +2 and +1) close-up lens on our 70–300 mm lens to form a uniformly sharp image of the subject, seated 60 cm away from the camera.

Finally, the sensitivity of the system is greatly enhanced by eliminating visible light using an infrared (IR) filter that filters wavelengths below 850 nm, because the pupil tracking relies solely on infrared illumination.

### Illumination

Because the iris has a high reflectance in the IR range, IR illumination can be used to selectively visualize the pupil. Pupil tracking using IR illumination is by far the most common method used in commercial eye tracking systems. In addition to visualizing the pupil, the corneal reflection of the IR illuminator serves as an important cue to head translation and the two features—pupil and corneal reflection—can be combined to minimize the influence of small head movements on eye tracking accuracy (Holmqvist, 2015).

The most common source of problems in dark pupil tracking stem from illumination—either insufficient illumination that renders portions of the orbit as dark as the pupil and thus confuses the image segmentation, or temporal noise in the illuminator which totally disrupts the temporal precision of a tracker. Both challenges are overcome by using a large array of light-emitting diodes (LEDs) that emit only in the IR range with a stable power source.

Large arrays emit more light and do so more uniformly over space, thus overcoming the first challenge. LED's rely on direct-current (DC) power source and instability in the DC source (called ripple) can result in temporal noise. Ripples are common in all standard DC wall-plug adaptors, and these power sources are therefore inappropriate for eye tracking illumination. Ripples cause fluctuations in the light level and these fluctuations are more visible at high temporal frequencies (e.g., 490 fps), rendering high-speed tracking impossible. While it is possible to build an ultra-low ripple power source, we found that a desktop testing DC source—a common instrument in any electronics lab—provides for a perfectly steady DC source and results in negligible ripple. This low DC noise is also valuable for MEG, EEG, and single unit recordings as it minimizes the potential electrical noise in the recordings.

High intensity infrared illumination can be hazardous to eye health over continuous exposure. One paper suggests broadband infrared illumination in the range of 700–1400 nm is to be restricted to less than 1 mW/cm^2^ of corneal irradiance for a continuous exposure of 8 h (Mulvey et al., 2008), but a report from the COGAIN Association (Mulvey et al., 2008) could not determine a precise safety guide for bandpass illumination (i.e., 850 nm) and observed a wide range of illuminator intensities in commercially-available eyetrackers. We have measured the irradiance of our illuminator using a Graseby Optronics 5 (UDT Instruments, San Diego, CA) model S370 optometer using a Model #262 radiometric sensor with correction set to the illumination wavelength of our LED array (850 nm). The sensor measured 0.7 × 0.7 cm (~0.5 cm^2^), and we therefore scaled the measurements of power at 850 nm to estimate the power in cm^2^. At 20, 30, 40, and 50 cm distance from the illuminator, we measured 5.2, 3.2, 2.0, and 1.4 mW/cm^2^.

### Mechanical mount and control: motorized pan and tilt

Interaction with the lens for zooming and focusing is always needed during a recording setup. Such interaction will typically move the lens and camera, forcing the user to repeatedly adjust the setup until it is in focus and properly aligned with the subject's eye. We found that by using an off-the-shelf motorized pan and tilt system we were able to substantially accelerates and facilitates recording setup. This was particularly useful for applications on macaques as well as for humans in the MEG setup, where access to the inside of the MEG room during a scan is limited.

### Computer for tracking and broadcasting eye trace data

Our eye tracking system relies on CPU-based image processing. We found that an i7 Quad-Core 2 Ghz+ system with 4 GB of RAM was sufficient for processing the data in real-time with no frame loss. The system must be equipped with a USB 3.0 PCIe card (or built-in USB 3.0 slot) to enable the high-performance camera acquisition. The system operated under Windows 7 and interacted with the stimulus PC (a separate PC) using the TCP/IP interface described further below.

### Scene monitor

In many applications, such as animal behavioral training, it is desirable to view the eye trace over a copy of the display shown to the animal. Some vendors term this the “Scene Monitor” and we were able to implement this easily into our system. We first use a display splitter to get access to a real-time copy of the scene shown to the subject. This signal is then down-sampled using an external peripheral and converted to an analog video signal. Direct digital capture and transfer of HD-resolution displays makes extensive demands on the CPU and causes the system to slow down, while external down-sampling to VGA resolution allows the scene to be effectively represented for real-time visualization. The scene monitor is useful in any situation where the experimenter wishes to either monitor where the subject is looking in the scene, or to catch problems in calibration and tracking.

### Stimulus synchronization

Synchronization of events to the eye trace data is important for many types of neuroscience research. In many commercial trackers, synchronization is handled by the processing computer using either network events or an analog event signal. This approach introduces an inherent delay in the aligning the two signals (the external event and the eye traces), which can also become variable as a function of what else the CPU is doing. In our system synchronization is obtained by direct tagging of the camera image using an external TTL pulse—when the pin 1 of the GPIO connector of the Flea3 camera receives a square pulse with a voltage change greater than 3.3 V (i.e., a digital input), it records the state of this pin in the frame header of the image captured at that time point. Thus, the external event is sampled simultaneously with the eye image. The header information is then accessible in the recorded eye trace data for analysis of stimulus-driven oculumotor behavior.

### Software

Our software is based on the open-source GazeParser C and Python libraries 5 (Sogo, 2013) that can be controlled via Python. Our system incorporates a number of additions:

1. Graphical user interface (GUI) to quickly start the tracker and setup a recording file.
2. Modify the acquisition settings (pupil threshold, corneal reflection threshold, etc.) on the fly with GUI sliders.
3. Extensive calibration features, including a user-guided calibration procedure for minimally-cooperative subjects such as animals or infants.
4. Ability to re-use a calibration, facilitating psychophysical studies for cooperative and minimally cooperative subjects (i.e., restarting acquisition after a break or separate session).
5. Quadratic transformation of the eye-to-screen mapping for more uniform accuracy across the screen.
6. Scene monitor for monitor gaze position on the presented scene.
7. Recording of the external stimulus events with the eye timestamps.
8. Free recording of eye position in the absence of a stimulus using a click of a button.

## Materials

### Equipment

1. Camera: PointGrey Flea3 USB 3.0 FL3-U3-13Y3M-C, available from Point Grey Research Inc. (Richmond, BC, Canada; ptgrey.com for $595 USD).
2. Lens: Tamron 70–300 mm f/4-5.6 Di LD Macro Lens for Pentax AF, (Tamron # AF017P-700; sold at Bhphotovideo.com, TA70300MP for $199 USD). This is a Pentax K-Mount lens with a 62 mm filter thread.
3. Lens adaptor: General Brand C-Mount Adapter for Pentax K Lens (sold by B&H Photo, catalog # GBCMP; $29.95 USD). This adaptor is needed to connect the Tamron lens (Pentax K-mount type) to the camera (c-mount type).
4. Close-up lenses: HOYA close-up lens kit for 62 mm filter thread, including +1, +2, +4 (MFR # B-62CUS-GB; sold by B&H Photo, Catalog # HOCUS62 for $35.00 USD).
5. Infrared Filter: 850 nm Infrared IR Pass Filter for 62 mm filter thread, sold by Deal Extreme (Dx.com, SKU 22986, sold for $23.50 USD).
6. Illuminator: 96 LED array, consisting of 850 nm LEDs (sold by Deal Extreme, dx.com, sku# 41108, sold for $32 USD).
7. Illuminator Mount: Oben TT-100 Table Top Tripod (sold by B&H Photo, bhphoto.com sku OBTT100, for $34.95).
8. Power supply: DC regulated power supply for steady illumination. HY3003D-2-R from Jameco Electronics (www.jameco.com, sold for $219.95 USD).
9. GPIO Cable for stimulus time stamping: 8 pins, 1 m GPIO Cable, Hirose HR25 Circular Connector, available from Point Grey Research Inc. (ptgrey.com, sku ACC-01-3000, sold for $35 USD).
10. Photodiode for stimulus synchronization (Optional): Hamamatsu S7610-10 photodiode with a built-in amplifier and Schmitt trigger. (available from Hamamatsu Inc. for $5 USD each).
11. A 1.5 k Ohms 5% resistor available from any electronics supplier. The color bands for such a resistor are brown, green, red, and gold, in that order.
12. USB 3.0 interface card: Although the built-in USB 3.0 interface may suffice in many cases, Point Grey Research Inc. recommends using the Fresco FL1100, 2 Port, USB 3.0 Host Controller Card which can be purchased from Point Grey Research (ptgrey.com sku U3-PCIE2-2P01X sold for $60 USD).
13. USB 3.0 cable: we recommend using the locking USB 3.0 sold by Point Grey Research Inc. (available from ptgrey.com, sku ACC-01-2300, sold for $10 USD). This cable provides screws to securely attach to the camera.
14. Camera Mount: Bescor motorized pan and tilt mount with remote control, monted on a mini-tripod. The entire kit consists of the motor head (Bescor MP1K; sold by B&H Photo, sku # BEMP1K for $139 USD), the power supply for the motor (Bescor PS260, sold by B&H Photo, sku # BEPS260 for $16.50 USD), the remote control (Bescor RE20, sold by B&H Photo, sku # BERE20 for $14.95 USD), and miniature tripod (Bescor TH36, sold by B&H Photo, sku # BETH36 for $34.95 USD).
15. Processing computer: an Intel i7 computer with USB 3.0 and 8 gb of ram (e.g., Dell XPS 8900, sold by Dell.com for $849.99 USD).
16. Software: We recommend downloading our package (available from Farivarlab.com), which includes the following required sub-packages: our modified GazeParser package (Sogo, 2013), accessible from FarivarLab.com, MatPlotLib, NumPy, PIL, PyGame, Pyglet, PyOpenGL, SciPy, wxPython, and PySide. All libraries are licensed under the Open Source license and are free for download.
17. Optional chin rest: We recommend a chin-rest to maintain headposition during tracking (e.g., HeadSpot from Cambridge Research Systems, sku N2000, sold for £450).
18. Optional scene monitoring pipeline: We have used the Adesso AV-200 for composite video capture via USB (sold by B&H Photos, sku# ADAV200 for $29.99 USD). A lower-latency option is a PCI-based capture card such as the Hauppauge Impact VCB PCIe card (model 01381, sold by hauppage.com for $49 USD). These systems will digitize a scaled-down analog signal. This analog signal is obtained from the scene shown to the subject via a generic display splitter (either a VGA or a DVI splitter, based on the type of setup). The VGA or DVI input can be converted to a low-resolution composite video for low-bandwidth capture and display via a device such as the StarTech VGA-to-composite converter (sold by B&H Photo, bhphoto.com sku STVGA2VID for $83.75 USD) or the Atlona HDMI/DVI to composite converter (sold by B&H Photo, bhphoto.com sku ATHD530 for $349.99 USD). Additionally, a generic composite video cable (available at computer and Audio-Visual stores) is needed to connect the output of the converter to the input of the framegrabber.

### Procedure

#### Step 1–5 (CRITICAL) optical components and camera mount (Timing: 5 min)

1. Attach the IR filter to the zoom lens by inserting it into the front thread of the lens.
2. Attach the lens mount bracket to the c-mount adaptor, and then attach the lens via the c-mount adaptor to the Flea3 camera.
3. The camera has a standard tripod screw hole at the bottom, which must be mated directly onto the motorized pan-tilt system.
4. The pan-tilt motor also has a screw hole for a tripod thread and must be attached to the min-tripod using this screw.
5. Make sure all screws are tight to minimize vibrations during acquisition.

#### Steps 6–19 (CRITICAL) electrical and data connections (Timing: 30–60 min)

6. Attach the Besco power supply to the motorized pan-tilt system, and connect the Besco remote control to the main motor.
7. Attach the GPIO connector to the camera, and then connect the USB 3.0 cable to the camera.
8. To synchronize eye trace with the stimulus onset, connect the Hamamatsu photodiode to the camera. The camera GPIO has 8 wires (pins). The three relevant wires for the diode connection are pins 1, 6, and 8, corresponding to the black, blue, and yellow wires of the GPIO connector. Pin 1 is the input connection for the receipt of the TTL pulse, Pin 6 is the ground connection, and Pin 8 is a 3.3 V electrical output supply. The Hamamatsu photodiode has 3 pins, numbered from left to right looking directly at the sensor. On the photodiode, Pin 1 (leftmost) is the ground, Pin 2 (middle) is the TTL output, and Pin 3 (rightmost) is the power supply input.
9. To mate the photodiode to the GPIO connector, simply connect the grounds together (blue wire on GPIO to middle pin of the photodiode).
10. Connect the power supplies together (yellow wire of GPIO to the rightmost connector on the photodiode).
11. Connect the TTL output of the photodiode to the input of the GPIO connector (black wire on the GPIO connector to the middle pin of the photodiode).
12. Place a 1.5 k Ohms 5% resistor between the middle and the rightmost pin. Use a soldering iron for a permanent connection.
13. Place the photodiode facing the stimulus display at a bottom corner of the monitor (lower right or lower left) and fasten with tape or other mechanical means.
14. Disconnect the computer from its power supply, open the chassis and insert the dedicated PCIe USB 3.0 card into a free port, following the manufacturer's instructions.
15. Make sure to attach a motherboard power connector (a 4-pin power connector) to the USB 3.0 card, because the camera receives its power directly from the PC. Note that in the absence of free motherboard power supplies, it is possible to directly power the camera using the GPIO cable.
16. After closing the computer, attach the USB cable from the camera to the newly-inserted USB 3.0 card and power up the computer.
17. To install the PCIe framegrabber to enable to scene monitoring, install the Hauppage ImpactVCB card following the manufacturer's instructions. The USB 2.0 framegrabber (Adesso framegrabber) simply needs to be plugged into a free USB 2.0 port.
18. Depending on the output of the stimulus presentation computer (VGA, DVI or HDMI), attach a splitter to duplicate the output. The duplicated output must then be converted to a composite video signal using the appropriate converter (see above).
19. The down-sampled analog video is then subsequently digitized by the framegrabber and must be connected to it using a standard composite video cable.

#### Steps 20–26 (CRITICAL) illuminator setup (Timing 30–60 min)

20. The illuminator LED array is as standard CCTV night-vision light source and is supplied with an automatic light sensor. This light sensor is easily removed to allow for direct control of the LED array. Open the LED housing by unscrewing the single screw at the bottom.
21. Remove the sensor by using a hot soldering iron or small cable cutters to break and remove the sensor.
22. To attach the illuminator to the power supply, first cut the tip of the cable using a cable cutter, and then proceed to strip open the cable using the fine blade of a box-cutter.
23. Strip the two small single cables and wind them around the high and ground connections of the DC regulated power supply.
24. Ensure that the power supply is set to only 12 V maximum—more than this amount can burn the LEDs. Once the voltage is set to 12 V, adjust the current dial until the LEDs illuminate (visible through the camera or observable directly by the emission of faint red light).
25. The illuminator is ready for use and can be positioned either by using the supplied bracket or by attaching the housing to a ball jointed desktop tripod for greater flexibility. The illuminator should be located under you stimuli display and not in the same optical angle as the camera where it would create a bright pupil image.
26. For this latter approach, a ¼” hole must be drilled into the bottom of the illuminator housing approximately 1.5 inch (4 cm) away from the face of the array.

#### Steps 27–35 (CRITICAL) software setup (30–60 min)

27. The camera requires the 32-bit Flea3 drivers available from ptgrey.com. These must be downloaded and installed on the system.
28. In addition, the high-speed USB 3.0 acquisition requires a modified USB 3.0 driver also available for download from the ptgrey.com website.
29. Following the manufacturer's instructions, install the appropriate driver for the camera and the USB 3.0 interface.
30. The camera installation can be verified by running the FlyCapture software provided in the driver package.
31. To install the eye tracking software, download the compressed package from our website (FarivarLab.com), following the included instructions.
32. If the scene monitor is also desired, install the software for the scene monitoring framegrabber (either the USB or PCIe system).
33. Ensure the correct operation of the scene monitor by running the manufacturer-supplied viewer software.
34. To synchronize the stimulus onset with the eye tracking, turn the pixels facing the photodiode to white on the same frame as your stimulus and black otherwise. This simple protocol can be easily implemented by drawing a white box in the bottom corner of the screen corresponding to the photodiode. This white box is to be drawn on the same frame as the main stimulus. After the stimulus is removed, set the pixels covering the photodiode to black. This is the most accurate means of synchronizing the display to the eye trace since it uses the screen refresh on which the stimulus was shown to tag the camera frame acquired in that moment.
35. It is optionally possible to synchronize another event (e.g., pulse from a brain stimulation device or other external device) with the eye trace using the GPIO connector. In these cases, only pins 1 and 6 are needed to be connected to the TTL output and ground of the external device, respectively.

#### Steps 36–47 (CRITICAL): starting the system and setting up tracking

36. Navigate to the eye tracking software directory and double-click on the SimpleGazeTracker_Flea3.exe to start the tracking core.
37. Double click on the MVR GUI to open the graphical user interface to control tracker functions.
38. In the “Connection” panel, enter the filename for the output tracking data files. The IP field can be set to the IP of the tracker PC, or unchanged if the GUI is being used on the tracker PC.
39. Click “Connect to SimleGazeTracker.” You should see a message appear informing you of a successful connection.
40. Once connected to the tracker, the user can move the sliders to control the tracking behavior of the system via the sliders.
41. Adjust the pupil threshold until only the pupil is filled with a blue video overlay.
42. Increase the size of the Purkinje search area so as to include the corneal reflection (bright spot on the eye).
43. Adjust the Purkinje threshold (corneal reflection) until only the corneal reflection is selected.
44. Reduce the Purkinje exclude area so that the overlap with the pupil image is minimal.
45. In the case of cooperative subjects, ask the subject to look at points on the screen that reflect the maximum range needed for the study.
46. In the case of minimally-cooperative subjects, we recommend designing salient stimuli (e.g., moving insects) and moving them over the screen so as to assess the range of eye positions.
47. Ensure that the parameters selected for steps 33–36 are adequate for the entire range of eye positions needed for your study.

#### Steps 48–56 (CRITICAL): creating, storing, and loading calibrations

48. Click on “Create Calibrate Settings” and then specify a name for your calibration file. This calibration file can be loaded in the future to facilitate setup, particularly for minimally-cooperative subjects.
49. By default, our system implements a quadratic transformation that requires a 9-point minimum calibration procedure (the “small” option). For less accuracy but faster setup, one may use the linear (5-point) calibration. For more precise estimate of gaze position, especially with cooperative subjects, you may use the “medium” or the “large” options. These built-in calibration options are based on an HD resolution screen (1920 × 1080) but they can be easily adjusted by modifying a text file.
50. Click “Next (Point Selection)” to begin the calibration procedure.
51. Calibration consists of presenting stimuli at known fixed positions on the screen and then mapping the pupil reflection and pupil center vector (originally in camera sensor coordinates) to the screen coordinates. Our system was designed for the more challenging minimally-cooperative subjects, such as macaques. In such a situation, the subject may not direct their gaze at the target stimulus at first, or may only hold their gaze there briefly. The user—while watching the eye image—can decide best whether the calibration point is being looked at or not and selecting the point that the subject is viewing on the Point Selection screen. Each button on this screen represents a location on the subject's screen where a stimulus will appear for the purpose of directing gaze during calibration.
52. Once the user is confident that the subject is directing their gaze at the stimulus, they can press the calibration button to confirm this and the button will turn green. In case of error, the button can be depressed and the point can be re-presented.
53. Upon completion of the 9-point procedure (for quadratic transform estimation), a text file is created in the “calib” directory.
54. In future sessions, this calibration file can be loaded by selecting “Load Calibration and Calibrate.”
55. Hint: With minimally-cooperative subjects such as macaques, it is impossible to motivate them to direct their gaze to a target without training. In naïve monkeys, we have found that loading calibration file obtained either from a human eye (in the same location) or another monkey was sufficient to obtain a coarse estimate of gaze position to allow for training the naïve animal to direct gaze toward a target and obtain reward. Once this training is established, we can then obtain a dedicated calibration estimate for that subject.
56. Once calibration is complete or loaded, recording can begin.

#### Steps 57–63 (CRITICAL): recording eye traces and monitoring in real-time

57. To simply start recording, click “Record” in the “Recording (Basic)” panel. This will begin storing eye trace data in the file specified in Step 30. (e.g., “%USERPROFILE%SimpleGazeTracker/file” on a Windows system).
58. Most researchers will want to send stimulus-specific information for inclusion with the eye trace data, such as condition name, stimulus number, and so on. For this, the tracking system can be remotely controlled by clicking on “Start Listening” in the “Recording (Advanced)” panel. Interactions with the core eye tracking software are managed by TCP/IP through the GazeParser interface (Sogo, 2013).
59. To monitor real-time eye position, click “Broadcaster ON” in the Broadcasting panel. This allows other software to obtain the eye position data in real-time. This information can be used for many purposes such as:
  - Behavioral reward: if gaze is directed toward a target stimulus, reward the subject
  - Gaze-contingent display: modify the display based on the position of gaze
  - Monitoring: Overlay the gaze position on the subjects' scene using the Scene Monitor
60. To enable real-time scene monitoring, launch the Scene Monitor executable from the eye tracking directory.
61. To facilitate viewing during fast eye movements, select “Trailing” to allow for multiple eye points to be plotted at the same time.
62. To stop remote monitoring, click the “Broadcaster” button so that it displays “off.”
63. To stop recording in the GUI, unclick “Record” in the Recording (Basic)” panel.

### Troubleshooting

**Table:** 

**Step**	**Problem**	**Possible reason(s)**	**Solution**
9	Camera does not operate	The USB 3.0 port does not have sufficient power	a. attach the motherboard power supply to the USB 3.0 controller Power the camera directly via the GPIO connector using power supply from Point Grey Research Inc.
19	Illuminator does not turn on	The LED array is wrongly wired.	Reverse the connecting wires for the LED array to the regulated power supply
31	Connection to SimpleGazeTracker fails	Drivers are not properly installed. The wrong drivers are installed	Ensure that you have only downloaded 32-bit versions of all software. Ensure that the FlyCapture2 software supplied by Point Grey Research Inc. can connect to the camera. If not, re-install following manufacturer's instructions
33	The pupil cannot be separate from other dark points in the image	Lighting is poor, creating dark regions other than pupil. An object may be obstructing the view of the eye.	Bring the illuminator closer. Adjust the illuminator to improve uniformity of light on subject's eyes and surrounding regions. In the case of animals, make sure hair is not obstructing the eye.
33	Pupil is perfectly detected during setup but lost during experiment	Pupil adjustments were made without accounting for testing light levels	Ensure that all light sources (ambient room lighting, screen light, etc.) are at the settings to be used during the experiment. Adjust your stimuli background color to be same as calibration background if possible (gray).
34-36	Multiple bright spots are confused as the Purkinje image	Other light sources are creating bright spots on the eye. Glare on spectacles is confused for corneal reflection	Dim ambient light in the room. Ensure that only one light source illuminates the eye. If possible remove jewelry and glasses causing the glares.
33-36	Pupil and Purkinje image are detected and tracked, but the trace is noisy	Light source is unstable. The optics or camera is unstable. Other light sources are interfering	Ensure that the light source is powered by a *regulated* DC power supply. Provide conditioned AC power to the DC power supply in case of wiring in older buildings. Ensure that the optics are securely fastened to the camera and that the camera is securely fastened to the tripod and motor. Ensure the camera and optics do not vibrate. Dim or turn-off ambient light sources.

### Timing

Steps 1–5: 5 min
Steps 6–19: 30–60 min
Steps 20–26: 30–60 min
Steps 27–35: 30–60 min
Steps 36–47: 10–30 min
Steps 48–56: 3–10 min, depending on subject cooperation
Steps 57–63: <1 min.

## Anticipated results

By following this protocol, the user should be able to track eye movements at high speeds (+450 fps) and with high-precision (up to 0.12° resolution) and tightly synchronized with stimulus onsets. We measured the stability of eye traces during fixation to a dot target that appeared at 1° intervals on a grid and moved every 0.5 s. This measurement naturally confounds the precision of the hardware with the biological noise around fixation, so we compared the precision of the system on average across all fixations to the most stable fixation epoch across the 49 fixations. The MVR Tracker rendered a precision of 0.12°. The reader will note that accurate measurement of the eye tracker precision requires use of an artificial eye (Poletti and Rucci, 2016). We plan to carry out a more thorough evaluation of the MVR Tracker precision and accuracy with an artificial eye, but the measurements we report for human subjects are likely attainable by others carrying out this protocol properly.

**Figure 3:**
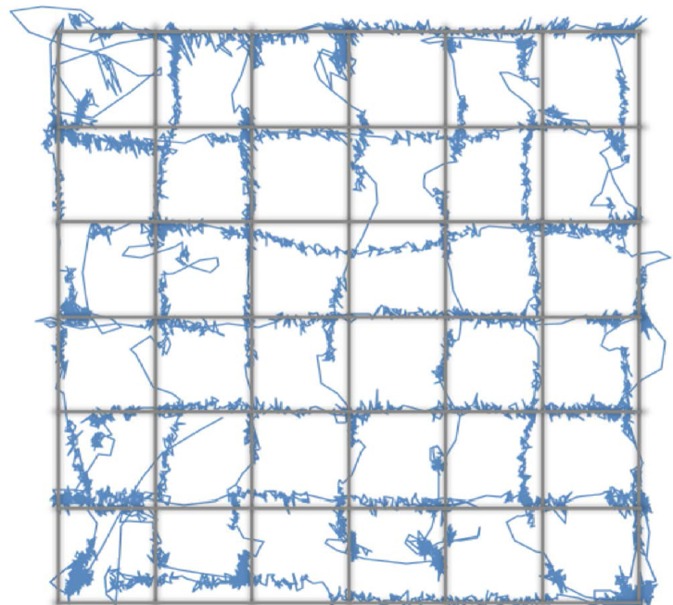
**The accuracy of the MVR tracker**. In this task, the subject viewed a dot moving along the grid lines with a sinusoidally-modulated velocity. This smooth pursuit task allowed us to test the spatial accuracy of the system and the calibration. While higher number of calibration points will result in better accuracy, our results demonstrate that with a 9-point calibration and with a quadratic mapping model, a highly accurate measure of gaze position can be made.

Because the system uses standard SLR lenses that are larger than the camera sensor, the system's accuracy and precision is uniform over the entire range of eye movements tracked during normal screen-viewing tasks (Figure 3). Here, we asked subjects to track a dot moving with a sinusoidally modulated velocity across the screen, tracing a grid with 1° × 1°cell elements. This test assesses the accuracy of the short 9-point calibration for mapping the gaze (initially in camera coordinates) to the screen coordinates. As can be seen in the eye-drawn grid, the accuracy of the system is high, with the largest deviations from the grid dominated by oculomotor errors. The precision of the system is sufficient to track these deviations. Note that with a greater than 9-point calibration even better accuracy can be achieved.

The user can optionally use the eye tracker output in real-time to control the stimulus (gaze-contingent displays) or to receive the subject's decision on a task such as is the case during training of macaque monkeys. We measured the real-time latency of the system using a digital feedback loop—while tracking the eye, we read the broadcasting eye positions using a TCP/IP link to the tracker through Matlab, and each time the eye position crossed a threshold amount (arbitrarily set to 100 pixels), Matlab triggered a TTL pulse on a LabJack U3 device. This TTL pulse was fed back into the eye tracking camera. Because each frame is timestamped at the time of acquisition, and because the eye position data is stored in relation to that timestamp, we compared the timestamp for when the eye position crossed our defined threshold to the timestamp when the TTL trigger was registered by the camera. We made this measurement while operating the camera at 250 fps and at 490 fps. At 250 fps, we observed a latency that matched the camera's latency—mean latency of 3.96 ms and standard deviation of 0.07 ms. At the higher speed, the latency increased to a mean of 4.99 ms and standard deviation of 0.18 ms, likely due to the increased computational overhead at the higher framerate. This short latency means that eye information can be available within a refresh rate of up to 200 Hz.

The system broadcasts the gaze position, blinks, and stimulus visibility (set by external TTL pulse) and the broadcast can be read by any software that supports UDP or TCP/IP communication protocols (e.g., Matlab, LabView, and most stimulus presentation software). These broadcasted signals can be easily used to make reward decisions for training animals or for other gaze-contingent displays. We routinely use this real-time broadcast of the gaze position to gauge the animal's decision during a four-alternate forced choice visual task.

## Author contributions

RF designed the eye tracking hardware, identified the software libraries, and defined the software development goals. DM developed all the software features including GUI, time-stamping, and non-linear mapping for calibration.

## Funding

Startup funds from the RI-MUHC and an NSERC Discovery Award (RGPIN 419235-2013) to RF.

### Conflict of interest statement

The authors declare that the research was conducted in the absence of any commercial or financial relationships that could be construed as a potential conflict of interest.
